# Perinatal Breathing Patterns and Survival in Mice Born Prematurely and at Term

**DOI:** 10.3389/fphys.2019.01113

**Published:** 2019-08-30

**Authors:** Sanja C. Ramirez, Jenna E. Koschnitzky, Tiffany M. Youngquist, Nathan A. Baertsch, Charles V. Smith, Jan-Marino Ramirez

**Affiliations:** ^1^Center for Integrative Brain Research, Seattle Children’s Research Institute, Seattle, WA, United States; ^2^Department of Neurological Surgery, University of Washington School of Medicine, Seattle, WA, United States

**Keywords:** prematurity, inflammation, breathing, respiratory rhythm, development, lipopolysaccharide, apnea, premature birth

## Abstract

Infants born prematurely, often associated with maternal infection, frequently exhibit breathing instabilities that require resuscitation. We hypothesized that breathing patterns during the first hour of life would be predictive of survival in an animal model of prematurity. Using plethysmography, we measured breathing patterns during the first hour after birth in mice born at term (Term 19.5), delivered prematurely on gestational day 18.5 following administration of low-dose lipopolysaccharide (LPS; 0.14 mg/kg) to pregnant dams (LPS 18.5), or delivered on gestational day 18.7 or 17.5 by caesarian section (C-S 18.5 and C-S 17.5, respectively). Our experimental approach allowed us to dissociate effects caused by inflammation, from effects due to premature birth in the absence of an inflammatory response. C-S 17.5 mice did not survive, whereas mortality was not increased in C-S 18.5 mice. However, in premature pups born at the same gestational age (day 18.5) in response to maternal LPS injection, mortality was significantly increased. Overall, mice that survived had higher birth weights and showed eupneic or gasping activity that was able to transition to normal breathing. Some mice also exhibited a “saw tooth” breathing pattern that was able to transition into eupnea during the first hour of life. In contrast, mice that did not survive showed distinct, large amplitude, long-lasting breaths that occurred at low frequency and did not transition into eupnea. This breathing pattern was only observed during the first hour of life and was more prevalent in LPS 18.5 and C-S 18.5 mice. Indeed, breath tidal volumes were higher in inflammation-induced premature pups than in pups delivered *via* C-section at equivalent gestational ages, whereas breathing frequencies were low in both LPS-induced and C-section-induced premature pups. We conclude that a breathing pattern characterized by low frequency and large tidal volume is a predictor for the failure to survive, and that these characteristics are more often seen when prematurity occurs in the context of maternal inflammation. Further insights into the mechanisms that generate these breathing patterns and how they transition to normal breathing may facilitate development of novel strategies to manage premature birth in humans.

## Introduction

Birth is associated with the abrupt and complex task of reconfiguring the respiratory system in order to establish and maintain adequate oxygenation and ventilation as soon as the baby is born. Perhaps not surprisingly, breathing often fails upon delivery, and 10% of the more than 100 million infants born annually worldwide require resuscitation (Wiswell, 2011). Infants requiring delivery room intubation and CPR have higher risks of death and/or neonatal complications than infants who manage the transition themselves (Jiang et al., 2016). Respiratory movements do exist *in utero*, but these fetal movements have very different functions than the ventilatory movements in air, and the fetal respiratory movements do not simply continue as the infant transitions into the radically different extrauterine environment. The first air breaths need to provide not only sufficient oxygenation, but also help to clear the lungs of fluids, and be of sufficient depth to open the alveoli and avoid atelectasis. Several human studies have revealed that the first breaths after birth are very deep (Karlberg et al., 1960; Karlberg and Koch, 1962; Mortola et al., 1982; Te Pas et al., 2009). Newborn babies and in particular preterm infants showed very long expiratory breath hold patterns (Te Pas et al., 2009). The so-called “braking mechanism” of expiration is thought to preserve functional residual capacity, which is important because the chest walls of newly born infants are very compliant (Te Pas et al., 2009). During the braking of expiration, the closed glottis, in combination with an increased intrapulmonary pressure, maintains airway pressures above atmospheric. Braking of expiration can occur in form of grunting or crying (Fisher et al., 1982; Te Pas et al., 2009). An open question is whether these breath hold patterns constitute a unique transitory breathing pattern that is specifically generated during the first minutes after birth.

Relatively little is known about the neuronal determinants that govern the transitions of breathing following birth in infants, particularly in those born prematurely. Understanding these determinants in preterm babies holds high clinical significance. Worldwide, 15 million babies are born preterm every year, which is associated with significant morbidity, mortality, and socio-economic burdens (Harrison and Goldenberg, 2016). Many human preterm births and stillbirths are associated with maternal infections (Romero et al., 2007; Goldenberg et al., 2008). Intrauterine infections also are associated with increased rates of preterm delivery and with poor neonatal outcomes, including low Apgar scores, admissions to neonatal intensive care units, neonatal mortality, lower gestational ages at birth, and lower birth weights (Adams-Chapman and Stoll, 2006; Moss, 2006; Kemp, 2014). Experimental animal models of premature births have been studied, based upon administration to pregnant animals of infectious agents or sterile fragments, such as lipopolysaccharide (LPS) (Kohmura et al., 2000; Guo et al., 2013). Although fetal and intrauterine infections clearly are capable of inducing intrauterine fetal demise, stillbirth, and premature delivery, the administration of LPS to pregnant mice was shown to depend upon maternal inflammatory responses and not upon LPS crossing the placenta to evoke fetal responses (Kohmura et al., 2000).

Although the results of some studies of early breathing patterns in human newborns have been published (Fisher et al., 1982; Mortola, 2019), human examples of early breathing are not readily amenable to experimental manipulation, as will be required to elucidate critical control mechanisms. We therefore examined the effects of LPS administration to pregnant mice, first on preterm delivery and short-term survival of the newborns, then on the breathing patterns and transitions of the pups. We compared the early breathing patterns of mice delivered at term to control dams with the patterns exhibited by mice delivered prematurely by caesarian section and with mice delivered prematurely in response to administration of LPS to dams. The goals of these studies were to determine whether mouse pups born prematurely would exhibit breathing abnormalities similar to those observed in premature human infants and whether these abnormalities might be exacerbated by maternal inflammation. In doing so, we identified breathing patterns, distinct from normal eupnea and gasping that were only expressed during the first hour of life. One of these patterns, characterized by very large and long-duration breaths was predictive of death and was most prevalent in pups born prematurely in response to maternal inflammation.

## Materials and Methods

### Animals

All methods and procedures were approved by Seattle Children’s Research Institute’s IACUC and done in accordance with NIH guidelines. C57BL/6 mice were group housed on a 12:12 light-dark cycle with access to chow and water *ad libitum*. Female and male mice were group housed until breeding. In house, timed pregnant mice were obtained by placing a male mouse in a harem at 5 pm and separating the male mouse from the female mice by 10 am the next morning. Female mice were checked for sperm plugs. If a plug was present, the female mouse would be housed separately.

### Manipulations

Four treatment groups were studied: (1) term mice that were born naturally on gestational day (G) 19.5; preterm mice born *via* cesarean section on either (2) G18.5 or (3) G17.5, 1 or 2 days preterm, respectively; (4) LPS-induced preterm mice born on G18.5.

### Lipopolysaccharide Injections

To generate the lipopolysaccharide (LPS) 18.5 group, LPS (EMD Millipore: Calbiochem, lipopolysaccharide *E. coli* O111:B4, lot # D00144477) was injected i.p. (0.14 mg/kg, dissolved in 0.1% sterile saline for a final concentration of 1 mg/ml) to pregnant female mice on gestational day (G) 17.5 at 5 pm. This dose of LPS was chosen based on previous reports using LPS to induce premature birth (Guo et al., 2013). Once in solution, LPS was sonicated and then stored as a frozen stock. Dams for Term 19.5 and C-S 18.5 groups received control injections of saline (5 μl) on G17.5 at 5 pm.

### Cesarean Section

Pregnant females at G17.5 or 18.5 were euthanized *via* cervical dislocation without anesthesia. A horizontal cut was made across the abdomen. The uterine horns were removed, and the pups were quickly dissected out of the uterus and embryonic sac. A paper wipe was used to dry the pup and remove excess fluid from the mouth. Six pups were removed from each female. The first 2–3 pups were used for subsequent breathing measures during the first 10 min of life.

### Survival Assessment

All pups were removed from the mother immediately after birth and placed on a circulating water pad set to 32°C. A cardboard box top with holes cut into the side was placed over the pups. Survival was assessed during the first 2 h after birth. Death was defined by cessation of breathing and failure to respond to tactile stimulation.

### Plethysmography

Breathing was recorded within the first 10 min after birth. Each recording lasted for 3–5 min. The C-S 17.5 group was not included due to difficulty in getting the pups to breathe repeatedly. Breathing patterns were recorded from unrestrained mice by whole-body, flow, barometric plethysmograph (flow: 60 ml/min; compressed air 21% O_2_, 0.1% CO_2_). Changes in pressure were measured between the experimental and reference chamber (both 60 ml) using a BUXCO pressure transducer and preamplifier. The signal was digitized at 1,000 Hz (Digidata 1322A, Molecular Devices, Sunnyvale, CA) and captured using Axoscope 10.3 data acquisition software (Molecular Devices). Data were analyzed off-line using Clampfit 10.3 software (Molecular Devices). The ambient chamber temperatures were maintained at 32°C (Physitemp TCAT-SDF Controller, Clifton, NJ). However, other factors that influence gas volume measurements such as body temperature, barometric pressure, and relative humidity were not accounted for in calculations of tidal volume. Thus, breath volumes were normalized to calibrations made during each recording using a 50-μl Hamilton syringe attached to the experimental chamber. The amplitude ratio of breaths compared to calibrations was used to determine a value in microliter for each breath. To account for differences in body size, breath volumes were also normalized to body weight (μl/g). Although we acknowledge the limitations of plethysmography for precisely measuring tidal volume, our conclusions are primarily based on the relative size of breaths between experimental groups, not precise volumes. As a result, we do not expect these limitations to have a significant impact on the interpretation of our data.

### Data Analysis

Data were analyzed and graphed using GraphPad Prism 6 software. Data are presented as means±SE and statistical significance was determined at *p* < 0.05. Significance is indicated by: ****p < 0.0001, ***p < 0.001, **p < 0.01, and *p < 0.05. Appropriate *t*-tests (with or without Welch’s correction) or one- or two-way ANOVA with Bonferroni corrected *post hoc* tests were used to compare groups. Repeated measures ANOVA were used where appropriate. Details of each comparison and factors used for each two-way ANOVA are provided in figure legends.

## Results

### Inflammation and Prematurity Influence Survival

**Figure 1:**
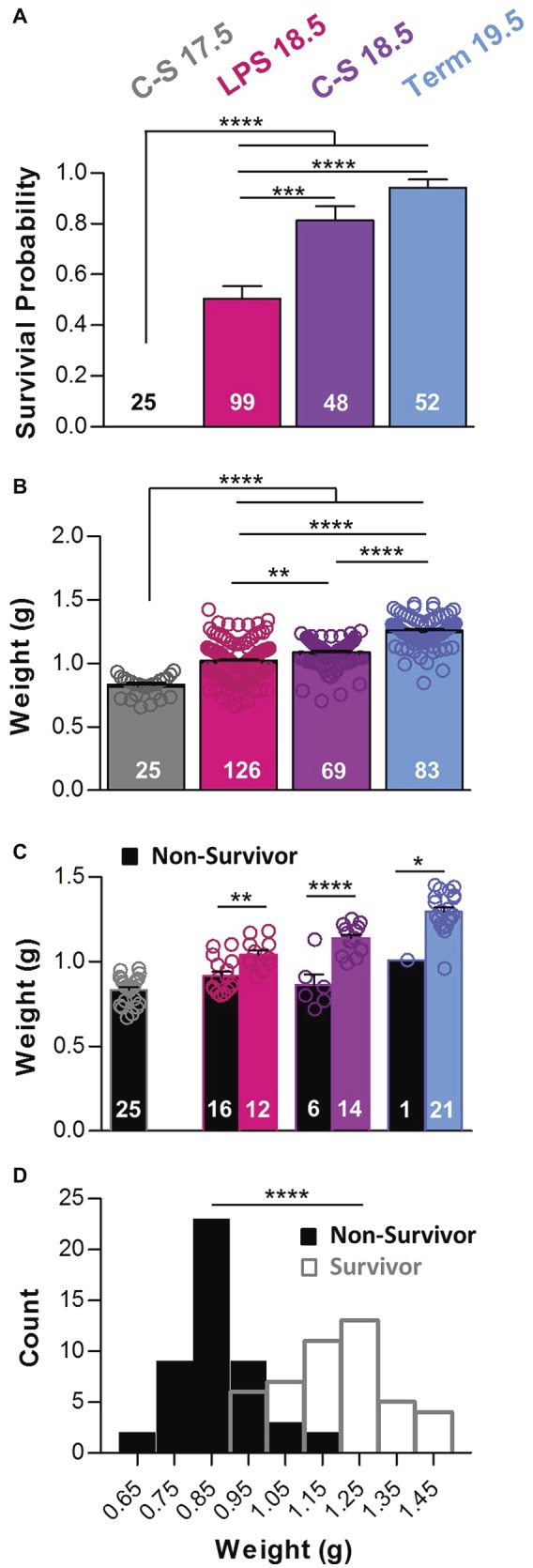
Prematurity, inflammation, and birth weight influence survival. **(A)** The probability of survival within 2 h after birth was different among groups (One-way ANOVA; *p* < 0.0001). *Post hoc* comparisons with Bonferroni corrections show that percent survival was lower in the C-S 17.5 group than in all other groups and lower in the LPS 18.5 animals than in both the C-S18.5 and Term 19.5 groups. **(B)** Pup weights also differed with manipulation (One-way ANOVA; *p* < 0.0001). Weights increased with gestation age and were lower in the C-S 17.5 animals than in all other groups and in the LPS 18.5 and C-S 18.5 groups than in the Term 19.5 group. **(C)** Weights were lower for pups that died (black bars) than for pups that survived (colored bars) for the LPS 18.5, C-S 18.5, and Term 19.5 groups (two-way ANOVA; *p* < 0.0001; factors: treatment group and survival). **(D)** Pooled frequency distributions from all groups showing combined birth weights of survivors (open bars) were significantly higher than for non-survivors (black bars) (two-tailed *t*-test with Welch’s correction).

We compared the survival rates among neonatal mice that were born naturally on G19.5, mice born 1 or 2 days prematurely *via* cesarean section, or mice born vaginally 1 day prematurely due to maternal inflammation. Survival rates during the first 2 h after birth were different across manipulations (Figure 1A). Most mice born naturally at term on G19.5 (Term 19.5; *n* = 52) survived (94%). Mice delivered prematurely by cesarean section (C-S) on G18.5 (C-S 18.5; *n* = 48) also exhibited a high survival rate (81%), close to those of term mice (*p* > 0.05), indicating that 1 day of prematurity alone is not a major contributor to mortality in these animals. However, survival of mice born prematurely on G18.5 in response to maternal administration of low-dose LPS on G17.5 (LPS 18.5; *n* = 99) was 50%, which was lower than both the C-S 18.5 and Term 19.5 groups (*p* < 0.001) suggesting that the inflammatory response contributes significantly to the greater rate of mortality in these pups. A fourth group of mice delivered *via* C-S on G17.5 (C-S 17.5; *n* = 25) exhibited survival of 0%, which was lower than the LPS 18.5, C-S 18.5, and Term 19.5 groups (*p* < 0.0001). Survival was not different between male and female mice in any group (two-way ANOVA with Bonferonni *post hoc* tests, *p* > 0.05). In the Term 19.5 group, 92% of males and 96% of females survived. In C-S 18.5 mice, 78% of males and 84% of females survived. And in LPS 18.5 mice, 62% of males and 48% of females survived. Thus, sex was not predictive of survival in these premature mice.

### Body Weight at Birth Is Related to Survival

In general, younger pups weighed less than older pups (Figure 1B). C-S 17.5 pups (*n* = 25) weighed 0.83 ± 0.02 g, which was less than LPS (*n* = 126) and C-S 18.5 pups (*n* = 69) (1.02 ± 0.01 g and 1.09 ± 0.01 g, respectively), as well as Term 19.5 pups (*n* = 83; 1.26 ± 0.01 g; *p* < 0.0001). The weights of LPS 18.5 and C-S 18.5 pups were both different from Term 19.5 pups (*p* < 0.0001). The weights of LPS 18.5 pups were also lower than C-S18.5 (*p* < 0.01), despite being the same gestational age. Within each treatment group, pups that died less than 2 h after birth weighed less than those that survived (Figure 1C). LPS 18.5 pups that died (*n* = 16) weighed 0.92 ± 0.03 g and those that survived (*n* = 12) weighed 1.04 ± 0.02 g (*p* < 0.01). C-S 18.5 pups that died (*n* = 6) weighed 0.86 ± 0.06 g and those that survived (*n* = 14) weighed 1.14 ± 0.02 g (*p* < 0.0001). Term 19.5 pups that died (*n* = 1) weighed 1.01 g, whereas survivors (*n* = 21) weighed 1.30 ± 0.03 g. Overall, newborns that failed to survive (*n* = 48; 0.87 ± 0.01 g) for 2 h weighed less than did those that survived (*n* = 46; 1.18 ± 0.02 g; *p* < 0.0001) (Figure 1D).

### Breathing Patterns After Preterm Birth

**Figure 2:**
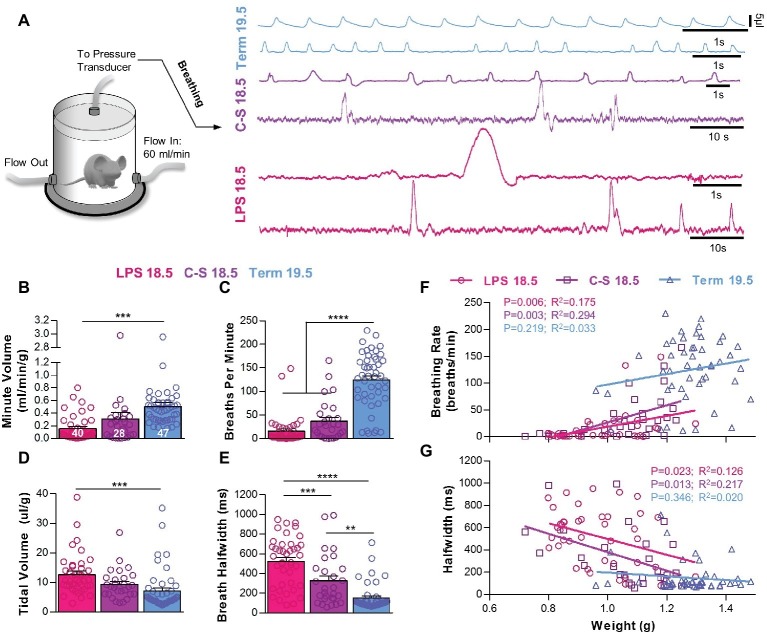
The influence of prematurity and inflammation on breathing parameters during early life. **(A)** Diagram of the whole-body, flow plethysmograph apparatus (reference chamber for the plethysmograph is not shown) and two representative traces of breathing activity in each experimental group. Quantified breathing patterns within 5 min of birth showing **(B)** minute volume, **(C)** breathing frequency, **(D)** tidal volume, and **(E)** breath half-width. All breathing parameters were significantly different among groups (one-way ANOVAs; *p* < 0.001). Relationships between birth weight and breathing rate **(F)** and breath half-width **(G)** were significant for premature LPS 18.5 and C-S 18.5 pups but not Term 19.5 pups. (linear regression analysis).

Breathing activities were measured using whole-body, barometric plethysmography, and representative recordings for each experimental group are shown in Figure 2A. Minute volumes were different across treatment groups. Minute volumes in LPS 18.5 mice (0.16 ± 0.03 ml/min/g), but not C-S 18.5 mice (0.31 ± 0.10 ml/min/g), were smaller than Term 19.5 mice (0.51 ± 0.06 ml/min/g; *p* < 0.001) (Figure 2B). This effect was primarily associated with slower breathing frequencies in LPS 18.5 (16.21 ± 4.79 breaths/min) and C-S 18.5 mice (37.03 ± 8.08 breaths/min) compared to Term 19.5 mice (124.7 ± 8.64 breaths/min; *p* < 0.0001) (Figure 2C). However, tidal volumes normalized to body weights in LPS 18.5 pups (12.67 ± 1.08 μl/g), but not in C-S 18.5 pups (9.37 ± 0.95 μl/g), were larger than in Term 19.5 pups (7.09 ± 1.00 μl/g; *p* < 0.001) (Figure 2D). The durations of individual breaths, measured as the peak half-widths (peak widths at half-height), were larger in LPS 18.5 pups (524.0 ± 39.0 ms) than in C-S 18.5 (328.2 ± 48.8 ms) and Term 19.5 pups (151.0 ± 19.6 ms; *p* < 0.001) (Figure 2E). Mice in the LPS 18.5 group (*n* = 40) therefore had very low breathing rates with large amplitude and long-duration breaths, whereas Term 19.5 mice (*n* = 47) had more frequent and smaller breaths. C-S 18.5 mice (*n* = 28) were generally intermediate, suggesting that premature termination of intrauterine fetal development and the presumed maternal inflammation responses invoked by administration of LPS combine to influence breathing patterns observed in the newborn pups. Breathing patterns also varied with body weights. Lower weights were related to slower breathing rates in LPS 18.5 (*p* < 0.01) and C-S 18.5 mice (*p* < 0.01), but not Term 19.5 mice (*p* > 0.05) (Figure 2F). Breath half-widths were also significantly related to weight in LPS 18.5 (*p* < 0.05) and C-S 18.5 mice (*p* < 0.05), but not Term 19.5 mice (*p* > 0.05) (Figure 2G). Thus, breathing patterns were related to birth weights only when pups were born prematurely.

**Figure 3:**
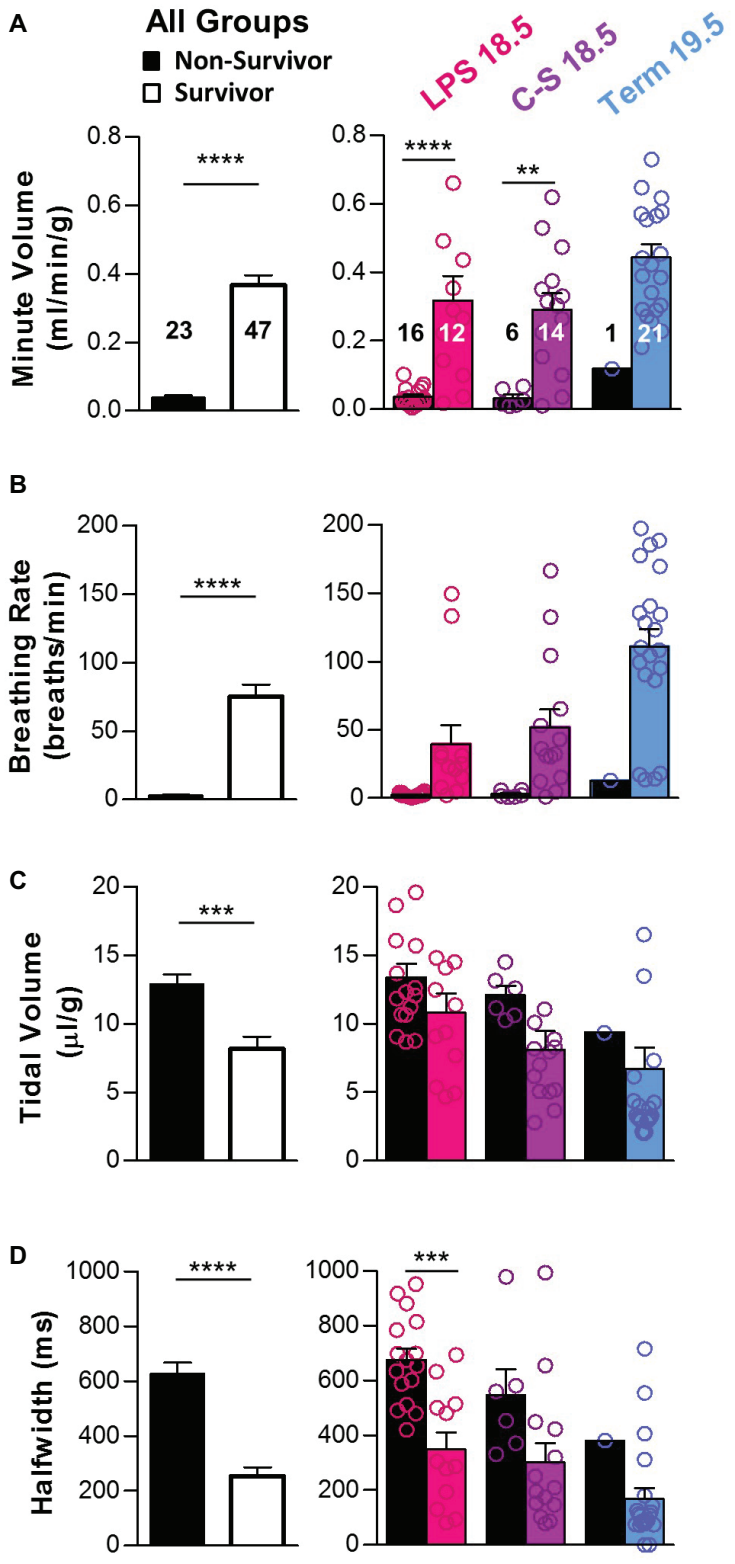
Breathing parameters during early life as predictors of survival. Breathing parameters were quantified in pups that survived the first 2 h of life (colored bars) and compared to those that did not (black bars). **(A)** Overall, minute volume was significantly higher in pups that survived (two-tailed *t*-test with Welch’s correction). Differences within each treatment group (LPS 18.5, C-S 18.5, and Term 19.5) are shown on the right (two-way ANOVA; *p* < 0.0001). **(B)** Overall, breathing rate was also higher in pups that survived (two-tailed *t*-test with Welch’s correction). Differences within each treatment group are shown on the right (two-way ANOVA; *p* < 0.001). **(C)** Overall, tidal volume was lower in pups that survived (two-tailed *t*-test with Welch’s correction), but differences were not detected within each treatment group (two-way ANOVA; *p* > 0.05). **(D)** Overall, breath half-width was larger in pups that survived (two-tailed *t*-test). Differences within each treatment group are shown on the right (two-way ANOVA; *p* < 0.01). Factors considered for all two-way ANOVAs were treatment group and survival.

Breathing parameters were also compared between mice that died (*n* = 23) within the first 2 h post-delivery and those that survived (*n* = 47) (Figure 3). Overall, mice that died had lower minute volumes (0.04 ± 0.01 vs. 0.37 ± 0.03 ml/min/g; *p* < 0.0001) (Figure 3A) primarily associated with slower breathing rates (2.93 ± 0.57 vs. 75.10 ± 8.98; *p* < 0.0001) (Figure 3B). Mice that died also had larger tidal volumes (12.90 ± 0.74 vs. 8.19 ± 0.89 μl/g; *p* < 0.001) (Figure 3C) and longer breath half-widths (628.8 vs. 254.4 ms; *p* < 0.0001) (Figure 3D) than those that survived. These trends in breathing parameters between survivors and non-survivors were apparent in all treatment groups.

Because mice breathe faster than humans, apneas are typically defined as the cessation of two or more respiratory cycles (Nakamura et al., 2003). This duration is considered to be equivalent to a pause of >10 s in humans (Davis and O’Donnell, 2013). However, the different breathing patterns reported here were characterized by strikingly different respiratory cycle lengths. For example, the “slow, large breathing pattern” often observed in LPS 18.5 mice was characterized by very long baseline cycle lengths, and therefore a 10-s interval would not be considered an apnea in these mice. Yet, this 10-s interval will certainly cause more hypoxia to the brain than an apnea of >two cycle lengths in a mouse breathing normally at 2 Hz. Thus, from this perspective, it seems that under baseline conditions, a typical LPS 18.5 mouse experiences an “apnea” almost every respiratory cycle. However, using different definitions of apnea for each distinct breathing pattern would seem arbitrary and confusing. Thus, in the present study, we present a careful quantification of breathing rate and other parameters without attempting to score the breaths or use different apnea definitions.

### Distinct Breath Types During Early Postnatal Life

**Figure 4:**
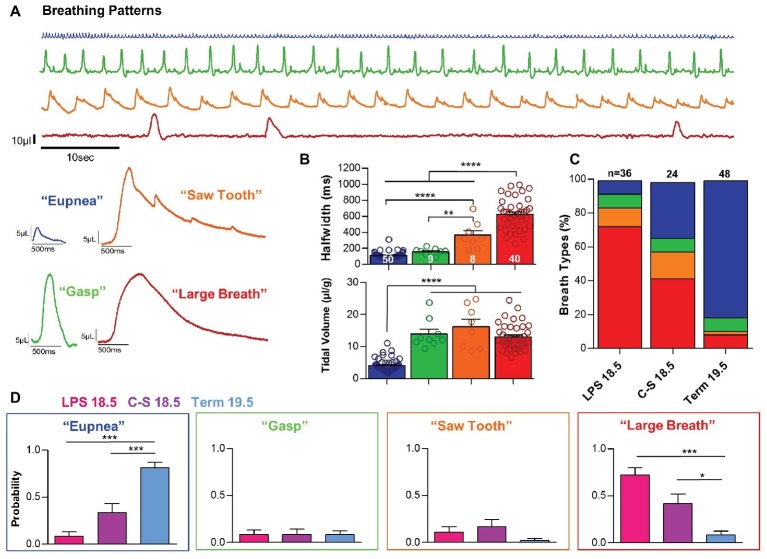
Inflammation and prematurity influence the prevalence of distinct breath types during early life. **(A)** Representative examples of the four breath types- “eupnea,” “gasp,” “saw tooth,” and “large-breath” that were observed during the first 10 min of life. **(B)** These distinct breathing patterns could be differentiated based on breath half-width (one-way ANOVA; *p* < 0.0001) and tidal volume (one-way ANOVA; *p* < 0.0001). **(C,D)** Quantified prevalence of each breath type within each experimental group: LPS 18.5, C-S 18.5, and Term 19.5. (one-way ANOVAs: Eupnea, *p* < 0.0001; Gasp, *p* > 0.05; Saw, *p* > 0.05; Large, *p* < 0.001).

The quantitative differences in breathing parameters described in the previous paragraph were associated with qualitatively different breath types. We observed four distinct patterns, which we refer to as “eupneic activity,” “gasping,” “sawtooth breaths,” and “large breaths” (Figure 4A). “Eupneic” or normal breathing activity (*n* = 50) was characterized by relatively high respiratory frequencies and inspiratory efforts with small half-widths (109.7 ± 5.9 ms) and small tidal volumes (4.05 ± .027 μl/g) (Figure 4B). Occasionally, we observed “gasps” (*n* = 9). This respiratory activity exhibited half-widths similar to those observed with eupneic breathing (153.0 ± 16.0 ms; *p* > 0.05), but gasps are differentiated from “eupneic activity” by the much larger tidal volumes (13.92 ± 1.50 μl/g; *p* < 0.0001) (Figure 4B). Gasps and eupneic breathing are also observed in mature mice (Baertsch et al., 2019) and are not specific to perinatal respiratory activities.

However, we also observed two types of activity patterns that were limited to the first hour after birth and are not observed in mature mice. Inspiratory efforts we refer to as “large breaths” (*n* = 40) were characterized by large tidal volumes (13.01 ± 0.64 μl/g), as well as very long half-widths (624.0 ± 30.3 ms) (Figure 4, red bars and trace). The long durations of these respiratory events clearly differentiate this pattern from gasps (*p* < 0.0001). Large breaths were typically associated with long pauses between breaths, resulting in very slow respiratory frequencies. Somewhat similar to large breaths were respiratory events that we refer to as “saw tooth pattern” (*n* = 8) (Figure 4, orange bars and trace). The tidal volume of saw tooth breaths (16.23 ± 2.29 μl/g) was similar to the large breaths (*p* > 0.05), and half-widths (363.3 ± 56.0 ms) were intermediate between gasps (*p* < 0.01) and large breaths (*p* > 0.05). However, sawtooth breaths have two characteristics that differentiate them from the large breaths. Most obvious is the superimposition of eupneic-like breaths that create the saw tooth pattern in the pressure curves. Secondly, the onset of these breaths is different from the large breaths. It appears that the onset may reflect a prolonged gasp in the saw tooth pattern, while in the large breaths, the onset is much rounder (Figure 4A, compare red and orange traces). Quantified tidal volumes and half-widths for each breath pattern are shown in Figure 4B.

All four breathing patterns were observed during the first hour after birth in both term and preterm mice. However, differences were observed in the prevalence of these patterns among groups (Figures 4C,D). During the first 10 min post birth, the probability of eupneic breathing was significantly greater in Term 19.5 mice (0.81 ± 0.06) than in LPS 18.5 (0.08 ± 0.05) and C-S 18.5 (0.33 ± 0.10) mice (*p* < 0.001). In contrast, the probability of large breaths was higher in LPS 18.5 (0.72 ± 0.08) and C-S 18.5 (0.42 ± 0.10) mice than Term 19.5 mice (0.083 ± 0.04; *p* < 0.05). Gasping and saw tooth breathing patterns were comparatively rare and their prevalence did not differ between term and preterm mice (*p* > 0.05).

### Early Postnatal Breathing Patterns and Survival

**Figure 5:**
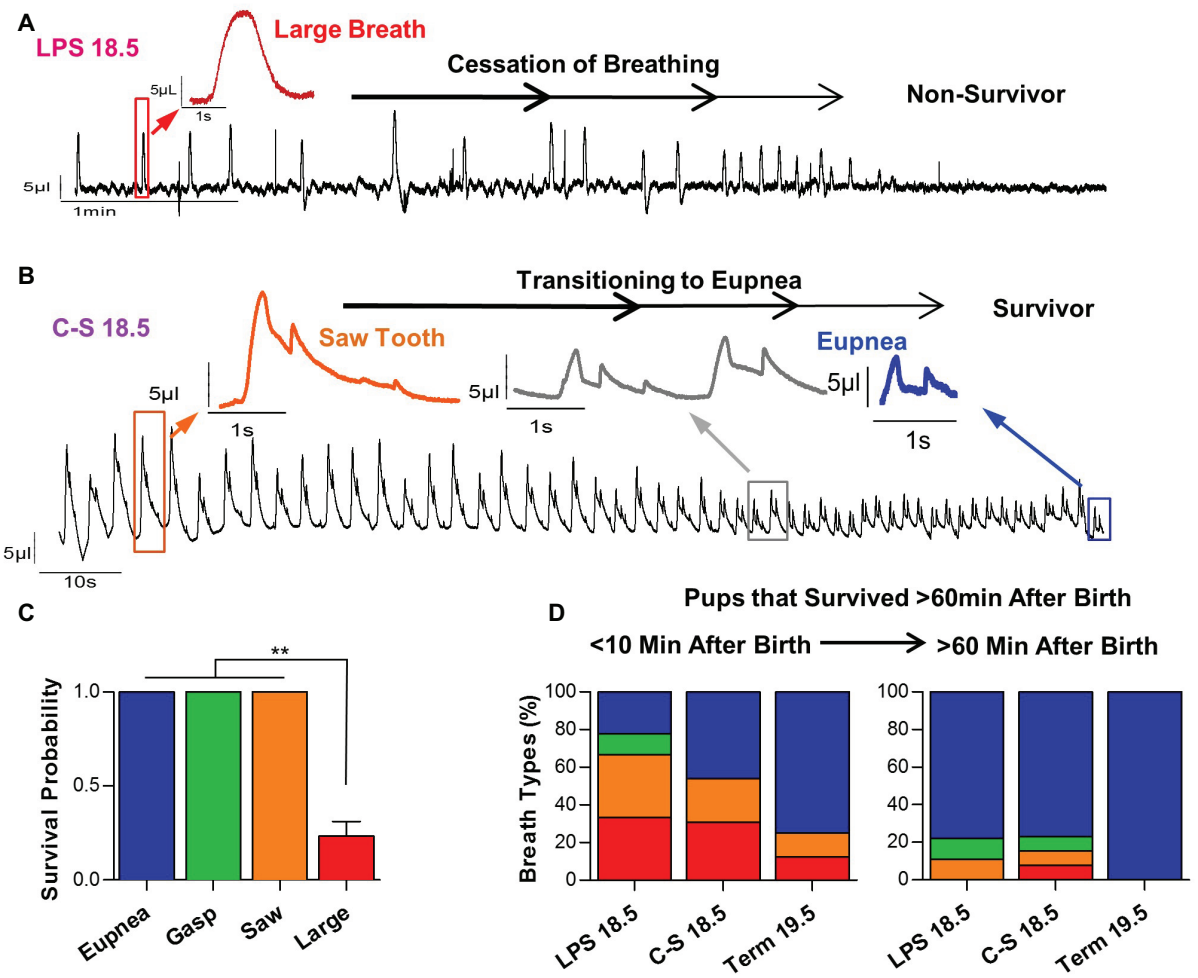
Relationships between early breathing patterns and survival. **(A)** Representative plethysmography recording of an LPS 18.5 pup that exhibits large breaths and dies following a failure to transition to a eupneic breathing pattern. **(B)** Representative plethysmography recording of a C-S 18.5 pup that exhibits a “saw tooth” breathing pattern initially, but successfully transitions to eupneic breathing and survives. **(C)** Pups that exhibited the “large breath” breathing pattern were much less likely to transition to normal breathing and survive (one-way ANOVA; *p* < 0.0001). **(D)** In pups that survive the first 60 min of life, “large breath” and “saw tooth” breathing patterns, which are more prevalent in LPS 18.5 and C-S 18.5 pups, successfully transition to a eupneic pattern.

These breathing patterns were predictive for survival (Figure 5). Pups that exhibited large breaths shortly after birth did not transition to normal breathing and did not sustain life. An example recording of an LPS 18.5 mouse with a large breathing pattern that fails to transition to normal breathing is shown in Figure 5A. Indeed, 100% of mice, term or preterm, that died within the first hour of life exhibited large breaths during the first 10 min after birth. Moreover, 67% of mice that exhibited large breaths (*n* = 30) during the first 10 min of life did not survive (Figure 5C). In contrast, preterm or term mice that exhibited either eupneic activity (*n* = 32), gasping (*n* = 2), or the saw tooth (*n* = 6) patterns survived the first hour of life after birth (*p* < 0.01) (Figure 5C). An example recording of a C-S 18.5 mouse with a saw tooth breathing pattern that successfully transitioned to eupneic breathing is shown in Figure 5B. Among pups that survived, breathing pattern transitions between the first 10 min of life and 1 h post birth are quantified in Figure 5D, demonstrating that saw tooth and large breath patterns can transition to eupnea to enable survival.

**Figure 6:**
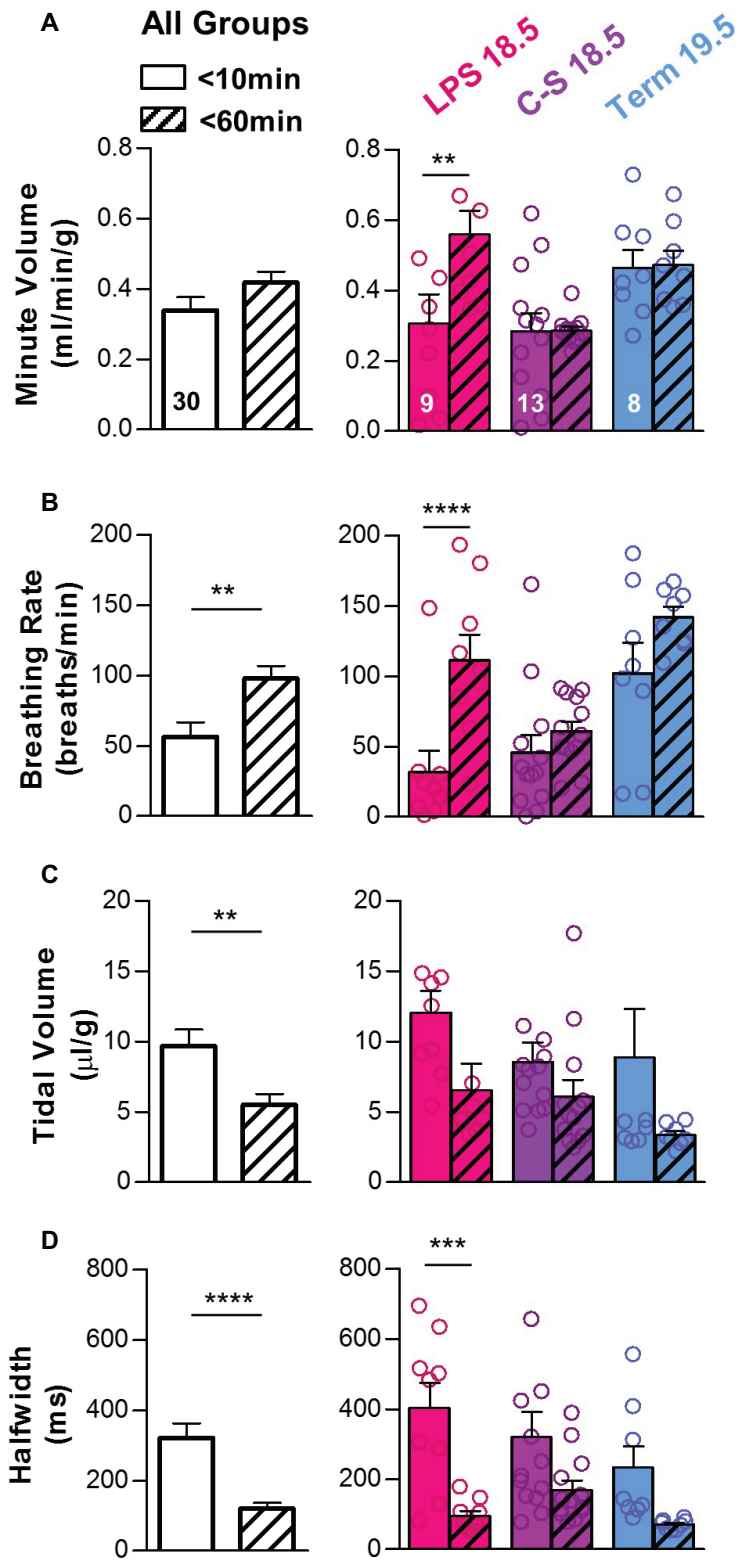
Changes in breathing patterns during the first hour of life. In pups that survived the first hour of life, breathing parameters were compared during the first 10 min of life (open bars) and at 60 min after birth (hatched bars). **(A)** Overall, minute volume was similar at both time points (two-tailed *t*-test), although LPS 18.5 pups that survived increased their minute volume. Differences within each treatment group (LPS 18.5, C-S 18.5, and Term 19.5) are shown on the right (RM two-way ANOVA; *p* < 0.05). **(B)** Overall, breathing rate increased during the first hour of life (two-tailed *t*-test), and this increase was most prominent in the LPS 18.5 group. Differences within each treatment group are shown on the right (RM two-way ANOVA; *p* < 0.0001). **(C)** Overall, tidal volume decreased (two-tailed *t*-test with Welch’s correction). Differences within each treatment group are shown on the right (RM two-way ANOVA; *p* > 0.01). **(D)** Overall, breath half-width decreased during the first hour of life (two-tailed *t*-test with Welch’s correction) and was most significant in the LPS 18.5 group. Differences within each treatment group are shown on the right (RM two-way ANOVA; *p* < 0.0001). Factors considered for all two-way ANOVAs were treatment group and time.

For pups that survived the first hour of life (*n* = 30), transitions in quantitative breathing parameters between the first 10 min of life and 1 h post birth are shown in Figure 6. Although overall changes in minute volume were small during the transition to normal breathing (0.34 ± 0.04 to 0.42 ± 0.03 ml/min/g; *p* > 0.05) (Figure 6A), breathing rates increased (56.6 ± 10.27 to 97.9 ± 8.8 breaths/min; *p* < 0.01) (Figure 6B), whereas tidal volumes (9.69 ± 1.18 to 5.51 ± 0.78 μl/g; *p* < 0.01) and breath half-widths (321.5 ± 41.6 to 121.0 ± 14.5 ms; *p* < 0.0001) decreased (Figures 6C,D). Thus, faster breathing with smaller breaths developed in pups that survived the first hour of life. Among LPS 18.5 pups that survived (*n* = 9), changes in minute volumes (0.31 ± 0.08 to 0.56 ± 0.07 ml/min/g; *p* < 0.01), breathing rates (32.09 ± 15.08 to 111.67 ± 17.96 breaths/min; *p* < 0.0001), tidal volumes (12.07 ± 1.55 to 6.55 ± 1.87 μl/g; *p* > 0.05), and breath half-widths (402.9 ± 71.3 to 95.0 ± 14.7 ms; *p* < 0.001) during this period were the most pronounced, suggesting that breathing must undergo the most dramatic changes during the first hour of life in LPS 18.5 pups in order to ensure survival.

## Discussion

Stimulating maternal inflammatory responses in pregnant mice leads to premature birth (Guo et al., 2013), low birth weight, respiratory disturbances, and early death, thus mimicking many of the detrimental consequences associated with premature birth in humans (Romero et al., 2007; Goldenberg et al., 2008). Our experimental approach allowed us to dissociate effects caused by the maternal inflammatory response from the effects of premature birth in the absence of inflammation. At the premature age of G18.5, mortality was not increased in mice delivered by C-section, when compared with term pups. In contrast, mortality was greater in pups born at the same gestational age but in response to maternal LPS injection. Lipopolysaccharides are endotoxins that trigger inflammatory responses in the absence of an actual bacterial infection. Studies with LPS-responsive C3H/HeN mice, hyporesponsive C3H/HeJ mice, and cross-bred pregnancies have shown convincingly that the maternal TLR4 status is the critical determinant of the effects of LPS in pregnant mice (Kohmura et al., 2000), with no effects observed in response to fetal receptor status. Thus, although we did not characterize the inflammation status of the pups, it is likely that the maternal inflammatory response (and not an actual infection) is the significant contributor to the observed mortality at this age.

In humans, low birth weights associated with, e.g., poor maternal lifestyle, gestational hypertension, intrauterine growth restriction, inflammation, pre-pregnancy obesity, and anorexia contribute to greater mortality (Papachatzi et al., 2013; Malin et al., 2014; Xaverius et al., 2014; Abubakari and Jahn, 2016). Various animal studies have also demonstrated that gestational stress and maternal separation are important contributors to the failure to thrive, which may have consequences for breathing function and the inflammatory response (Fournier et al., 2013, 2015; Battaglia et al., 2014; Soliz et al., 2016; Baldy et al., 2017). We expect that this form of neonatal stress contributed to all experimental groups, since all pups were separated from their mother immediately following birth. Our study found a relationship between birth weight and survival for all treatment groups. In general, mice with lower birth weights were more likely to die. Within each group the animals that died within 2 h after birth had lower body weights than those that survived. Thus, low birth weight is a significant predictor for mortality, independent of gestational age.

The data presented also demonstrate that inflammation-induced premature birth is associated with major respiratory disturbances. We found that prematurity is associated with low breathing rates and large tidal volumes, and this was exaggerated when prematurity occurred in response to maternal inflammation. Indeed, tidal volumes were higher in inflammation-induced premature pups than in pups delivered *via* C-section at equivalent gestational ages, indicating that maternal inflammatory responses contributed to the greater tidal volumes. By contrast, breathing frequencies were low in both LPS-induced and C-section-induced premature pups. This finding suggests that gestational age alone could produce low breathing frequencies and that factors that result in the very slow breathing rates may be related to the maturity of the central respiratory network, lung function, or breathing mechanics (Champagnat et al., 2011; Mellen and Thoby-Brisson, 2012; Alanis et al., 2014; Desai et al., 2014; Chevalier et al., 2016).

However, it is important to emphasize that within each experimental group there were mice that showed large-amplitude breaths with long pauses between them, and it was these mice that did not survive. All pups from all treatment groups that died during the first 60 min of life, exhibited large breaths. This was even the case in a small portion of mice born at term. These large breaths characterized by a long “braking pattern” had very different characteristics than normal eupneic breaths and gasps, suggesting that they are not just prolonged normal breaths or gasps, but instead may constitute a transitory breathing pattern that is characteristic of the first few minutes after birth. Future studies will be necessary to determine whether this transitory breathing pattern is generated at a distinct neuroanatomical site, has distinct neuromodulatory properties, and/or has distinct rhythm generating mechanisms when compared to the properties of eupneic activity, sighing or gasping. Indeed, it is even unknown whether this transitory pattern has a distinct adaptive functional role related to changes in lung function or breathing mechanics. One possibility may be the opening of alveoli in fluid-filled lungs right after birth. These large, long-lasting breaths may also serve as vocalizations to alert the mother. In human babies, cries and grunts are characteristic for the perinatal period. Irrespective of their potential functional role, our study demonstrates that these breaths are not capable of maintaining long-term survival. Mice that exhibited this large slow breathing pattern failed to transition to normal breathing during the first hour of life and died. This breathing pattern was more often seen in LPS-induced preterm than in C-section-induced preterm and term pups, suggesting maternal inflammation contributes substantially to breathing disturbances and mortality in these mice.

In contrast, mice that survived often showed large-amplitude and long-lasting breathing patterns that were superimposed by normal breaths. The simultaneous occurrence of large breaths and the small amplitude breaths generated at a fast, eupnea-like frequency resulting in a “saw tooth” breathing pattern in plethysmograph recordings. We propose that in these mice the “eupneic” breathing pattern was already present at birth, but occurred simultaneously with the large, long-lasting breathing pattern. Because eupneic breathing pattern was present at birth, these pups were able to transition to normal breathing once the large breathing pattern resolved. However, although much has been learned about the neuronal mechanisms that govern the generation of inspiratory and expiratory activity (Feldman et al., 2013; Anderson et al., 2016; Wu et al., 2017; Del Negro et al., 2018), and the control of breathing frequency (Baertsch et al., 2018), we have no mechanistic understanding of these long-lasting breaths.

We hope that this first report of unique perinatal breathing patterns will inspire further investigations into the neuronal substrates that regulate the transition to air breathing during the first minutes to hours of life. Follow-up studies may focus mechanistically on how the maternal inflammatory response suppresses the transition to normal eupneic breathing. We hypothesize that inflammation suppresses rhythm generation in the pre-Bötzinger complex, a critical network structure that is already functional at an early embryonic stage (Thoby-Brisson et al., 2005). Indeed, understanding the neuronal control that governs breathing transitions immediately following birth is an important goal for future investigation, and insights into how inflammation alters this neuronal control (Gresham et al., 2011; Ribeiro et al., 2017) may serve as a foundation for new strategies to overcome the need for artificial ventilation and to help guide the clinical management of infants when they are most vulnerable.

## Data Availability

All datasets generated for this study are included in the manuscript and/or the supplementary files.

## Ethics Statement

All methods and procedures were approved by Seattle Children’s Research Institute’s IACUC and done in accordance with NIH guidelines.

## Author Contributions

SR, JK, and TY acquired and analyzed data. SR, JK, J-MR, and CS conceived and designed experiments. SR, JK, NB, J-MR, and CS drafted and revised manuscript. J-MR approved final manuscript.

### Conflict of Interest Statement

The authors declare that the research was conducted in the absence of any commercial or financial relationships that could be construed as a potential conflict of interest.
